# Effect of different carbon allotropes for electrochemical glucose detection

**DOI:** 10.1007/s00216-026-06595-y

**Published:** 2026-06-06

**Authors:** Rui Couto, Joaquim Alves, Abel Duarte, Stefano Chiussi, Cristina Sousa, Felismina T. C. Moreira

**Affiliations:** 1 CIETI-LABRISE, ISEP, School of Engineering of the Polytechnic School of Porto, R. Dr. António Bernardino de Almeida, 431, 4249-015 Porto, Portugal; 2 CINTECX, Universidade de Vigo, 36310 Vigo, Spain

**Keywords:** Allotropes of carbon, Biosensor, Enzymes, Electrochemistry

## Abstract

The spread of point-of-care (PoC) glucose sensor technologies is crucial for effective diabetes management. In this context, we present a novel electrochemical biosensing platform that integrates various carbon allotropes—biographene (BGr), zinc oxide (ZnO)–modified multi-walled carbon nanotubes (MWCNT-ZnO), and biologically derived carbon dots (CDs)—in a polyaniline-based matrix (PANI) for glucose detection. The sensing layer was prepared by electropolymerizing aniline in the presence of the individual carbon allotropes and the enzyme glucose oxidase (GOx) on the surface of a screen-printed gold electrode (Au-SPE). The analytical performance of the sensor was evaluated by chronoamperometry (CA) in phosphate-buffered saline (PBS) and glucose-spiked human serum. The morphology and chemical composition of the carbon allotropes were characterized by using scanning electron microscopy (SEM) and X-ray photoelectron spectroscopy (XPS). Among the sensors tested, the carbon dot (CD)–based sensor demonstrated the best analytical performance, with a limit of detection (LOD) of 1.08 mmol/L and a sensitivity of 0.453 μA per decade of glucose concentration. This covers a linear working range of 1.56 to 75.0 mmol/L, which includes the typical physiological glucose concentration range observed in both healthy individuals and individuals with diabetes. This study aims to improve enzymatic glucose biosensors using fructose-derived carbon dots and PANI to enhance enzyme immobilization, electron transfer, and sensitivity while maintaining low cost and sustainability, offering strong potential for applications in personalized health monitoring and long-term chronic disease management.

**Supplementary Information:**

The online version contains supplementary material available at 10.1007/s00216-026-06595-y.

## Introduction

Diabetes mellitus is a chronic metabolic disorder characterized by persistent fasting hyperglycemia resulting from defects in insulin secretion, insulin action, or both. Early clinical manifestations are primarily related to sustained hyperglycemia and typically include classic symptoms such as polydipsia, polyphagia, polyuria, and blurred vision [1, 2]. According to the International Diabetes Federation, approximately 537 million adults aged 20 to 79 were living with diabetes worldwide in 2021, representing about 10% of the global adult population. This number is projected to rise to 643 million by 2030 and 783 million by 2045, driven largely by population aging and sedentary lifestyles associated with increasing urbanization [1, 2].

Traditionally, the diagnosis and monitoring of diabetes mellitus rely on laboratory-based tests such as fasting plasma glucose, the oral glucose tolerance test, and glycated hemoglobin measurements. Among analytical technologies, electrochemical biosensors are notable for their precise detection, selective response, rapid results, and affordable implementation [3, 4]. Electrochemical biosensors operate by converting a biochemical interaction, typically between glucose and an immobilized enzyme such as glucose oxidase (GOx), into an electrical signal that can be quantitatively measured. Their simplicity and portability make them especially attractive for point-of-care (PoC) testing, allowing individuals with diabetes to perform routine self-monitoring at home [5–10]. Electrochemical glucose biosensors have become essential tools for diabetes management since the first enzyme electrode was developed by Leland C. Clark Jr. and Champ Lyons in the early 1960s. These devices have evolved from oxygen-dependent systems to mediator-based platforms and, more recently, to direct electron transfer (DET) approaches [11–14]. Commercial devices, including blood glucose meters and continuous glucose monitors, primarily use enzymatic amperometric detection with optimized membranes and miniaturized electronics to provide real-time data for glycemic control [15]. However, challenges remain, including poor DET efficiency due to the buried redox center of GOx, enzyme instability, interference from electroactive species, biofouling during long-term monitoring, and variability in nanostructured electrodes [11–13].

To address these limitations, nanotechnology has significantly improved the performance of these biosensors. The integration of carbon-based nanomaterials such as graphene [16–18], carbon nanotubes [19–22], and carbon dots [23–25] into sensor platforms has shown great promise. Carbon nanostructures like graphene, carbon nanotubes, and carbon dots (CDs) have unique electrical, chemical, and surface properties that make them ideal for enhancing electron transfer and enzyme immobilization in biosensing systems [26]. By increasing electrical conductivity and expanding the electroactive surface area, these nanomaterials enable more efficient bioreceptor binding, which translates to lower limits of detection (LOD), faster response times, and greater analytical specificity [23, 27–30]. Consequently, carbon-enhanced devices are highly suitable for portable and wearable applications, allowing continuous and noninvasive monitoring—a major advancement over conventional techniques, especially for patients requiring intensive glycemic control.

In this context, extensive research has focused on exploiting the specific advantages of distinct carbon allotropes and their derivatives. Materials such as multi-walled carbon nanotubes (MWCNTs) [31], biographene (BGr) [32, 34, 35], and CDs [24, 25, 36, 37], along with hybrid composites like zinc oxide (ZnO)–modified MWCNTs [33], have gained significant attention. Their high surface-to-volume ratio, excellent electrical conductivity, and chemical stability make them ideal platforms for enzyme immobilization while facilitating the enhanced electron transfer crucial for optimal biosensor performance. For instance, incorporating semiconducting metal oxides like ZnO into MWCNT for electrochemical glucose sensors enhances functionality by promoting efficient charge transfer and creating a favorable microenvironment for enzyme attachment.

In ZnO nanoparticle–MWCNT architectures, GOx can be immobilized directly onto the ZnO surface without polymeric binders, while the MWCNTs act as primary conductive pathways. This synergistic configuration enhances current responses and analytical sensitivity, yielding reported linear detection ranges for glucose from 6.67 μM to 1.29 mM [38]. Similarly, ZnO nanotube–based biosensors use direct enzyme immobilization onto high-specific-surface-area ZnO nanotubular structures, to significantly increase enzyme loading and amperometric responses, with typical linear ranges of ~ 50 μM to 12 mM. However, relatively weak enzyme–ZnO interactions can negatively affect operational stability [39, 40]. Advanced ternary ZnO–graphene–MWCNT hybrid systems offer even superior electron transfer kinetics and amplified electrochemical signals, often achieving broader and more stable linear ranges, typically around 10 μM to 6.5 mM, depending on the composite architecture and enzyme loading parameters [41].

Beyond nanotubes and metal-oxide hybrids, graphene derivatives such as BGr provide a structurally robust and highly conductive matrix that ensures strong enzyme–support interactions. Regarding BGr/enzyme-based architectures, Alatzoglou et al. described for example a green production method for BGr, utilizing it as a support for the co-immobilization of three enzymes. The resulting biosensor demonstrated high stability, reusability, and efficiency in cascade enzymatic reactions, highlighting the potential of BGr for advanced sensing applications [42]. In PoC and self-powered platforms where BGr and GOx are entrapped in polymers, biosensors have achieved detection limits in the tens of micromolar range, though often with narrower linear ranges (approximately 0.1–5 mM) [35, 43].

Conversely, CDs—known for their biocompatibility and tunable electronic properties—offer a highly versatile strategy to further amplify electrochemical signals and increase detection sensitivity in enzymatic biosensors. Comparative studies of CD–polymer-based enzymatic glucose biosensors highlight that the polymer matrix is critical for analytical performance, working synergistically with CDs and GOx. Conductive polymers such as PANI and PEDOT/PEDOT:PSS-based composites generally provide the highest sensitivity, low LODs, and widest linear ranges due to their high conductivity and efficient charge transport, reflecting significantly improved CD-mediated electron transfer [44, 45].

CDs have also proven highly effective in non-enzymatic electrochemical glucose sensor based on copper oxide (CuO)–modified CDs, where the nanocomposite significantly enhances sensing performance. The sensor demonstrated excellent analytical performance, including a very low limit of detection (~ 1.4 nM) and a dual linear range of 15–225 nM and 0.1–0.85 mM, enabling detection from ultra-low to physiologically relevant glucose concentrations. The synergistic effect between CuO catalytic activity and CD electronic properties provided a highly sensitive and stable glucose sensing platform suitable for advanced biomedical applications [46].

Building upon the distinct electrochemical behaviors of these various carbon nanostructures, our work targets the evaluation of different carbon allotropes on electrochemical glucose detection while presenting a highly sustainable sensing strategy. Specifically, we embedded fructose-derived CDs in PANI to achieve high sensitivity with environmentally friendly, biocompatible, and low-cost materials. Using fructose as a carbon precursor provides a renewable, potentially waste-derived feedstock, aligning sensor fabrication with green chemistry principles and reducing reliance on petroleum-based or energy-intensive allotropes. The resulting CD–PANI composite offers abundant oxygenated groups, excellent aqueous dispersibility, and nanoscale dimensions, which enhance enzyme immobilization and facilitate electron transfer at the electrode. Furthermore, this approach also addresses the limitations of conventional carbon allotropes (such as graphene and carbon nanotubes), whose theoretical surface area is often inaccessible due to aggregation, bundling, and residual impurities. In contrast, the proposed CD–PANI platform is more scalable and reproducible, possesses a lower environmental footprint, and offers highly competitive electrochemical performance.

## Materials and methods

### Chemicals and solutions

Human serum was purchased from Cormay Diagnostics, D-(+)-glucose (dextrose monohydrate) from Alfa Aesar. Glucose oxidase HPS300 from *Aspergillus niger* was purchased from Sekisui Diagnostics; MWCNTs, zinc acetate and ethanol, diethanolamine, and phosphate-buffered saline (PBS) tablets from VWR. Creatinine and bovine serum albumin (BSA) were purchased from Fluka; uric acid (≥ 99% crystalline) from Sigma Aldrich; l-ascorbic acid from Riedel-De-Haen; aniline from Thermo Scientific; and urea from Fragor.

PBS at 10.0 mmol/L (pH 7.2) was prepared by dissolving a pellet of concentrated PBS in ultrapure water from the Milli-Q system. GOx enzyme solutions at 10.0 mg/mL and mixed solutions of GOx at 10.0 mg/mL with aniline at 10.0 mmol/L were prepared in PBS and applied to the electrodes. Glucose standards were prepared in both PBS and commercial human Cormay serum to generate calibration curves for each biosensor. A 100 mmol/L glucose stock solution was prepared in serum, and dilutions were made from this stock solution to obtain the following concentrations range within 0.19 to 100.0 mmol/L.

To evaluate selectivity, solutions with potential interfering factors were prepared in human serum. These included uric acid at 0.416 mmol/L, ascorbic acid at 0.085 mmol/L, creatinine at 0.115 mmol/L, and urea at 6.662 mmol/L. A glucose solution with 5.0 mmol/L in PBS was used as a reference to simulate physiological conditions. The concentrations of the interfering substances were adjusted to correspond to typical physiological values in human serum.

### Apparatus

The Au-SPE electrodes were purchased from Metrohm/DROPSENS and consisted of a working and counter electrode made of gold, along with a reference electrode made of silver. CV and CA were performed using a Metrohm Autolab potentiostat. A DropSens switch box was used to establish the connection between the electrodes and the potentiostat. Scanning electron microscope (SEM) analyses were carried out using a high-resolution scanning electron microscope, model JEOL JSM 6301 F, equipped with an Oxford INCA Energy 350 energy-dispersive spectrometer and a Gatan Alto 2500 detector.

X-ray photoelectron spectroscopy (XPS) measurements were performed on a Thermo Scientific K-Alpha ESCA instrument equipped with a monochromatic Al Kα source (hν = 1486.6 eV). Spectra were collected at a 90° take-off angle in constant analyzer energy mode, with pass energies of 100 eV (survey) and 20 eV (high resolution). Charge neutralization of the non-conductive samples was achieved using a low-energy electron flood gun (0–14 eV) in combination with a low-energy Ar+ ion gun. Binding energies were referenced to the C 1 s hydrocarbon peak at 284.8 eV, and elemental compositions were determined using Scofield photoemission cross-sections.

TEM analyses were performed using a JEOL JEM-1400 transmission electron microscope with STEM.

Chemical characterization was performed using a Thermo Scientific Fourier transform infrared spectrometer (Nicolet iS10) equipped with an attenuated total reflectance (ATR) accessory with a diamond crystal. Measurements were taken from 4000 to 500 cm⁻^1^ at 8 cm⁻^1^ resolution, averaging 120 scans each for the sample and background to improve the signal-to-noise ratio. Background spectra were recorded before each measurement to correct for atmospheric and instrumental effects, including H₂O and CO₂. Final spectra were collected in percent transmittance, and automatic atmospheric suppression was applied to reduce environmental interferences.

FTIR measurements used a background spectrum acquired from cellulose paper, as the carbon dots in colloidal suspension were incorporated into a cellulose matrix and then dried at 80 °C for 12 h to remove residual water.

### Synthesis of the allotropes of carbon

BGr was obtained by exfoliating graphite in the presence of BSA as described in reference [47], which facilitates dispersion of the material in water at room temperature using a blender. The procedure begins with a suspension of graphite crystals (100 mg/mL) in 200 mL of deionized water at pH 7.0 and BSA (3.0 mg/mL). For shearing, the suspension is processed in a blender for 30 min, with samples collected every 5 min to analyze the exfoliation rate. The absorbance of the suspension at 600 nm is measured by UV spectroscopy and used to quantify the graphene concentration after removal of unexfoliated graphite, which is separated by centrifugation at 1500 rpm for 45 min. In all exfoliation experiments, precise amounts of graphite, BSA, and deionized water are used.

ZnO-modified MWCNT composites were prepared as described in reference [48]. Zinc acetate dihydrate (0.97 g) was dissolved in 100 mL of absolute ethanol, followed by the addition of 5 mL of diethanolamine as a sol stabilizer. The mixture was stirred at 100 °C for 6 h. MWCNTs were then added to the stock solution at a concentration of 0.5% w/v. The MWCNT-containing sols were homogenized for 30 min at 6500 rpm using a homogenizer. Finally, the stock solution was heated at 100 °C for 12 h.

To obtain the CDs, an aqueous mixture was prepared by dissolving 1 kg/L fructose in water (solution A) and 6.8 g/L manganese(II) sulfate monohydrate in water (solution B). After both stock solutions were prepared, 2.5 mL of solution A and 2.5 mL of solution B were carefully mixed. To this mixture, 0.55 mL of concentrated sulfuric acid was added, followed by 15 mL of ethylene glycol. The mixture was heated in a household microwave oven at maximum power (750 W) with constant stirring for 60 s.

### Biosensing assembling

**Fig. 1:**
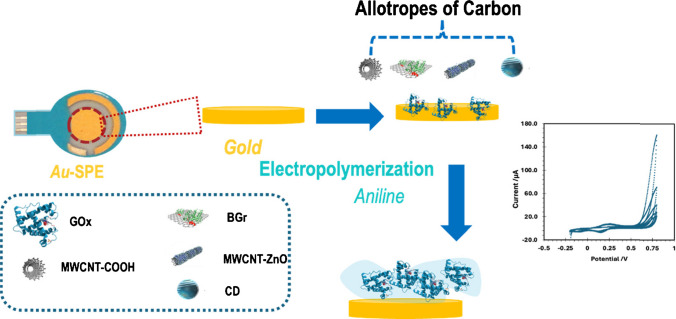
Schematic illustration of the construction of a glucose detection biosensor

Before electropolymerization (ELP), the Au-SPE electrodes were cleaned with ethanol and then electrochemically cleaned in 0.5 mol/L H_2_SO_4_ by applying cyclic voltammetry (CV) at potentials from − 0.2 V to 1.2 V for 5 cycles at a scan rate of 50 mV/s to activate the electrode surface. Composite films were produced by covering the working area of the Au-SPE with GOx mixed with a solution containing 0.01 mol/L aniline and nanomaterial precursors MWCNT-COOH, ZnO-modified MWCNTs, BGr, and CDs in PBS. The mixture was gently stirred to ensure homogeneous dispersion of the polymeric matrix (Fig. 1). The polymeric solution mixed with each carbon allotrope was deposited onto the electrode surface, and ELP was performed using a potential range of − 0.2 V to 0.8 V for 5 cycles. The process was conducted at a scan rate of 50 mV/s, allowing controlled film growth and enzyme entrapment within the polymeric network. After ELP, the modified electrode was carefully rinsed with PBS to remove any loosely bound enzyme or unreacted monomers.

### Electrochemical measurements

The oxidation peak of glucose on the enzyme-modified Au-SPE, representing the GOx-catalyzed reaction, was analyzed using cyclic CV. Measurements were performed over a potential range of −0.1 to +0.8 V at a scan rate of 0.01 V/s in both PBS solution (control) and 5.0 mmol/L glucose solution.

Biosensor performance was further evaluated by spiking glucose into PBS buffer and commercial human Cormay serum diluted 10- and 100-fold, enabling a linear correlation between current response and the logarithm of glucose concentration.

The calibration curve was obtained using chronoamperometry (CA) at a fixed potential of + 0.6 V, with standard glucose solutions ranging from 0.19 to 100.0 mmol/L in PBS buffer and from 0.78 to 100.0 mmol/L in diluted Cormay serum. For each measurement, the biosensor was incubated with 70 µL of the lowest concentration solution, and the current was recorded over 180 s with data acquisition every 0.1 s. The electrode surface was thoroughly cleaned between measurements before testing progressively higher glucose concentrations.

The limit of detection (LOD) was determined using the standard deviation of the blank (buffer) and the slope of the calibration curve, following established analytical guidelines. In this study, a semi-logarithmic calibration model was used, where the instrument response $\left(y\right)$ is linearly related to the logarithm of the analyte concentration $\left(x\right)$:

$$y=slope\cdot\log_{10}\left(x\right)$$

Selectivity assays were conducted by measuring the biosensor’s response in solutions containing 5.0 mmol/L glucose mixed with each of the following interferents: 0.416 mmol/L uric acid, 0.085 mmol/L creatinine, 0.115 mmol/L ascorbic acid, and 6.662 mmol/L urea. The CA technique was applied to each solution as previously described. This study was performed using the biosensor composed of the CD and a GOx layer composite within the PANI polymer matrix.

## Results and discussion

### Morphological characterization of materials

#### Transmission electron microscopy (TEM) analysis

**Fig. 2:**
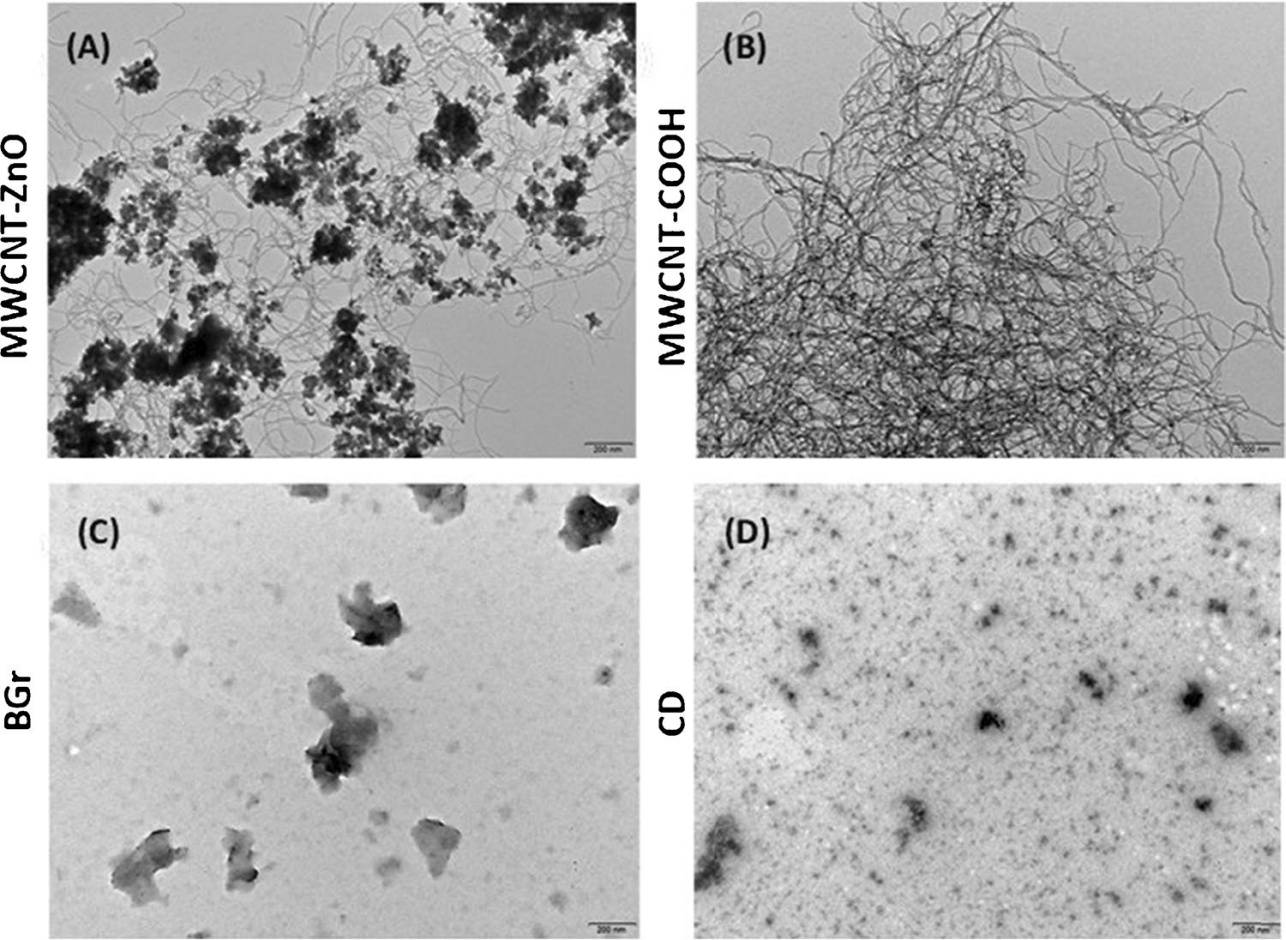
TEM analysis of MWCNT-ZnO (**a**), MWCNT-COOH (**b**), BGr (**c**), and CD (**d**) with magnification 50,000×

The TEM micrographs in Fig. 2 display distinct morphological features for each nanomaterial. In Fig. 2a, a compact, interconnected network of MWCNTs is visible, with dark agglomerated domains corresponding to ZnO nanoparticles distributed along the nanotube framework. The nanotubes are highly entangled, forming a conductive structure that is expected to enhance electron transport and increase the effective surface area. Figure 2b shows a more homogeneous and similarly entangled MWCNT network with fewer discernible aggregates, indicating improved nanotube dispersion. The tubular morphology remains clearly evident, confirming the preservation of the carbon nanotube structure after functionalization.

In Fig. 2c, sheet-like and irregular flake-like structures are visible, characteristic of graphene-based materials. Regions of higher contrast correspond to overlapping graphene layers or agglomeration, while thinner, more transparent areas are consistent with few-layer graphene domains. Figure 2d shows uniformly distributed nanoscale dark spots dispersed throughout the matrix, attributed to CDs. These particles have small nanometric dimensions and a homogeneous spatial distribution, indicating the successful synthesis of nanoscale carbonaceous entities.

#### Scanning electron microscope (SEM) analysis

**Fig. 3:**
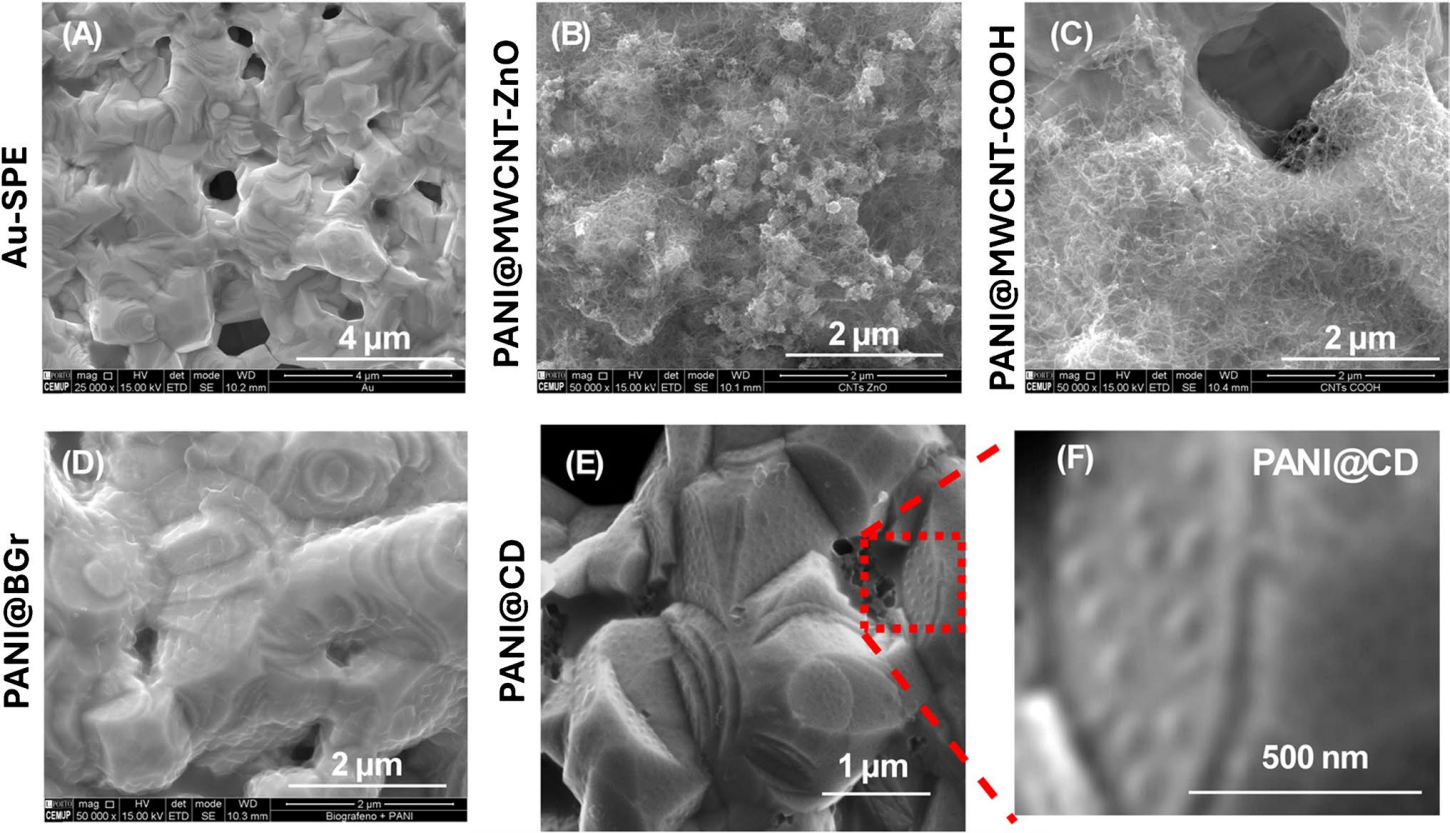
SEM analysis of Au-SPE (**a**), PANI@MWCNT-ZnO (**b**), PANI@MWCNT-COOH (**c**), PANI@BGr (**d**), and PANI@CD (**e**) with a zoomed region (**f**) to better visualize the CDs in the matrix

The SEM images provided valuable insights into the morphological characteristics of the samples, including the bare Au-SPE and four composite materials: PANI@MWCNT-ZnO, PANI@MWCNT-COOH, PANI@BGr, and PANI@CD (Fig. 3). The bare Au-SPE electrode has a relatively porous yet smooth surface **(**Fig. 3a). The PANI@MWCNT-ZnO composite **(**Fig. 2b) exhibits a rougher morphology, with a fine structure formed by the MWCNTs and ZnO nanoparticles. This increased surface area is particularly favorable for enzymatic biosensors, as it provides more active sites for interactions between the enzyme and glucose.

Both PANI@MWCNT-ZnO (Fig. 3b) and PANI@MWCNT-COOH (Fig. 3c) composites display entangled tubular structures with diameters of a few nanometers, consistent with multi-walled carbon nanotubes incorporated into the polyaniline matrix. The PANI@MWCNT-ZnO composite also shows a dense coverage of small, roughly spherical or granular particles uniformly distributed across the surface. These features indicate a nanostructured coating with significant surface area. The PANI@MWCNT-COOH composite displays a fibrous network consisting of both micro- and nanoscale fibers, forming an interlinked structure over the substrate. The 2-µm scale bar highlights the fine, interconnected morphology, which likely corresponds to functionalized carbon nanotubes or similar nanostructures. The PANI@BGr (Fig. 2d) composite, in contrast, exhibits the layered and sheet-like morphology characteristic of graphene-based materials. This structure provides high electrical conductivity, which facilitates charge transfer during enzymatic reactions and enhances the electrochemical response of the sensor. In the PANI@CD composite (Fig. 2e and f), uniformly distributed spherical nanoparticles with diameters of a few nanometers are observed, corresponding to carbon dots embedded within the polymer matrix. Composites with larger surface areas and rougher topographies, such as PANI@MWCNT-ZnO, PANI@MWCNT-COOH, PANI@BGr, and PANI@CD, provide abundant active sites for enzyme immobilization and effective interaction with analytes. These structural and surface properties are essential for enhancing the sensitivity and efficiency of biosensors in detecting low concentrations of target substances.

#### X-ray photoelectron spectroscopy (XPS)

**Fig. 4:**
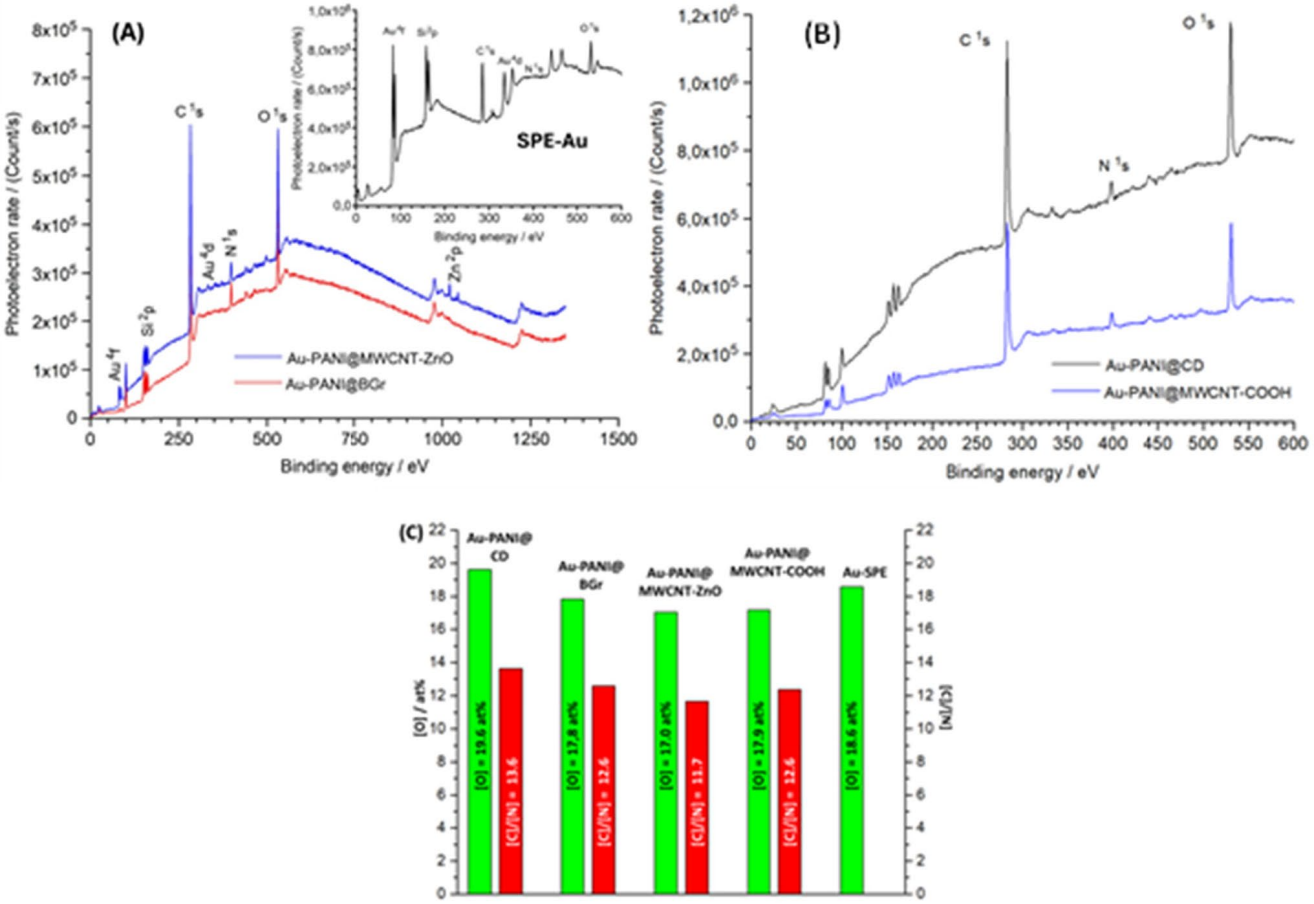
XPS spectra showing the binding energies in survey spectra of PANI@MWCNT-ZnO and PANI@BGr with the Au-SPE spectrum as inset (**a**), and PANI@CD with PANI@MWCNT-COOH (**b**), and a bar chart visualizing the oxygen content in at% and the C/N ratio for the evaluated spectra

The XPS analysis provided detailed insights into the surface composition and chemical states of the PANI-based composites. As shown in Fig. 4a, the atomic percentages revealed clear differences in oxygen content and the carbon-to-nitrogen (C/N) ratio among PANI@BGr, PANI@MWCNT-COOH, PANI@CD, and PANI@MWCNT-ZnO, compared to a bare commercial Au-SPE, included in this study as comparison. The XPS survey spectra of the composites clearly show characteristic peaks observed for carbon (C 1 s), nitrogen (N 1 s), and oxygen (O 1 s). Variations in peak intensities and shapes reflect different chemical environments of the composites and indicate different degrees of functionalization and interactions between bare Au-SPE and the PANI substrates with nanostructured functionalization. The bare commercial Au-SPE sensor surface produces stronger Au signals but also significant contamination with silicon, oxygen, and other elements, probably due to the ink used for producing the SPE sensors (inset of Fig. 4a). Adding the PANI composite clearly increases the carbon and nitrogen content as shown in Fig. 4a, as can be observed when comparing the bare Au-SPE with the PANI@MWCNT-COOH or PANI@CD survey spectra of the corresponding surfaces. On the other hand, incorporation of Zn by adding ZnO to the composite could be confirmed by the corresponding Zn peak in the PANI@MWCNT-ZnO spectra, as compared to the PANI@BGr ones (Fig. 4b). A distinct Zn 2p3/2 peak at approximately 1021 eV and a weaker Zn 2p1/2 peak at around 1045 eV are observed in the PANI@MWCNT-ZnO composite. Both peaks clearly stand out from the relatively noisy Auger background and are exclusively detected in the PANI@MWCNT-ZnO spectrum. A detailed analysis, using also the corresponding peaks through higher resolution spectra (0, 1 eV steps for binding energy), reveals that among the composites, PANI@CD exhibited the highest oxygen content (19.6 at%) and high C/N ration (13.6) followed by PANI@BGr with 17.8 at % oxygen content and a still relatively high C/N ratio (12.6), indicating the presence of numerous oxygen-containing functional groups and a larger proportion of carbon on the sensor surface. This behavior for PANI@BGr and PANI@CD can be attributed to the incorporation of graphene flakes that were exfoliated with BSA and for the high surface area and the wet chemistry production process of the CDs, which are known for their high surface functionality and oxygen-rich composition, thereby increasing the overall oxygen content. In contrast, the other composites presented slightly lower oxygen levels, ranging from 17.0 at% for PANI@MWCNT-ZnO, and 17.2 at% for PANI@MWCNT-COOH, together with lower C/N ratios between 11.7 (PANI@ MWCNT-ZnO) and 12.4 (PANI@MWCNT-COOH), compared to the PANI@BGr (Fig. 3c). These values suggest a comparatively lower density of oxygen-containing functional groups and a balanced or nitrogen-rich composition.

Overall, the XPS results demonstrate distinct surface chemistries among the PANI-based composites, with the PANI@CD composite notable for its higher oxygen content and C/N ratio. These differences highlight the influence of the incorporated nanostructured materials in tuning surface functionalization and interfacial interactions, providing valuable insights for potential applications and functionalization strategies.

### Performance of the GOx towards glucose oxidation

**Fig. 5:**
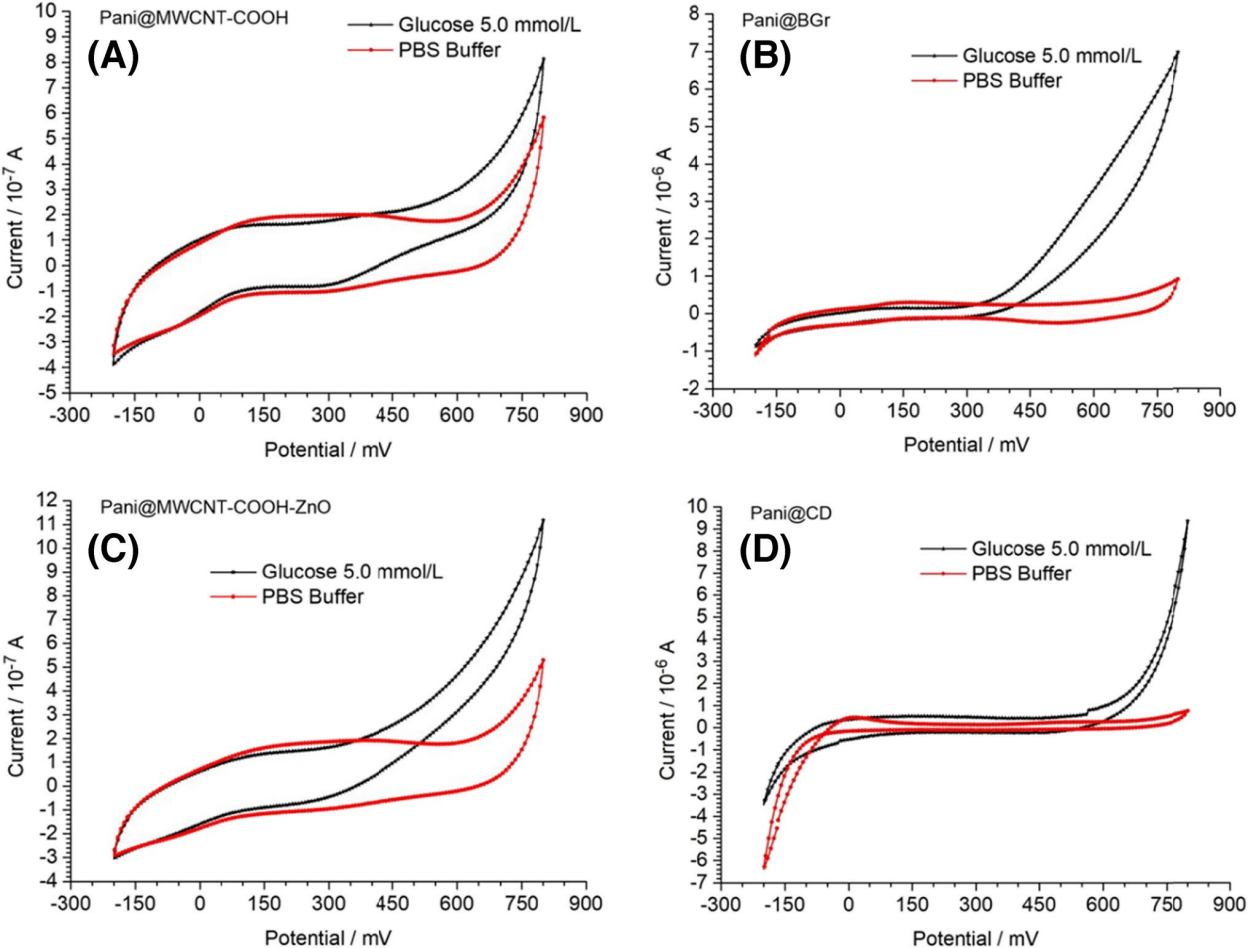
Voltammograms obtained in PBS and in a 5.0 mmol/L glucose solution at a scan rate of 0.01 V s^−1^. **a** Enzyme entrapped in a polymeric matrix of PANI@MWCNT-COOH; **b** PANI@BGr; **c** PANI@MWCNT-ZnO, and **d** PANI@CD

After assembling the biosensor using the ELP of aniline mixed with each carbon allotrope (Fig. 5), CV measurements were performed to evaluate the electro-oxidation of glucose catalyzed by GOx entrapped within the polymeric matrix. The electrocatalytic activity of the enzyme was assessed by comparing the electrochemical response in PBS, in the control, and in the presence of 5.0 mmol/L glucose, selected to mimic physiological conditions.

In Fig. 5a, the electrode modified with PANI@MWCNT-COOH shows a clear increase in current response when exposed to glucose, as it begins oxidizing hydrogen peroxide with oxygen serving as a mediator at + 0.4 V. This increase in signal demonstrates effective electrocatalysis, indicating that MWCNT-COOH enables efficient electron transfer between the GOx enzyme and the electrode surface. The performance is likely due to the high conductivity and large surface area of the nanotubes, as well as their good dispersion in the polymer matrix. Figure 5b shows different behavior for the PANI@BGr-based sensor. The presence of glucose also leads to a more pronounced increase in current after 0.35 V, with a prominent oxidation CV. This suggests that BGr supports enzyme immobilization and maintains GOx activity, and that the transfer efficiency is effective and depends on the morphology of the resulting film.

Figure 5c shows the response of the PANI@MWCNT-ZnO composite. Although the absolute current is lower than in the previous systems, there is a clear distinction between the buffer and glucose measurements. The incorporation of ZnO nanoparticles with MWCNTs may have a synergistic effect, providing structural support and improved enzyme stabilization, though with only a modest impact on conductivity. Nevertheless, the response remains clearly recognizable, confirming the functional activity of GOx in this hybrid material. Figure 5d presents the PANI@CD system, which displays the most prominent increase in current upon glucose addition, indicating a highly sensitive and efficient electrochemical interface. The significant separation between the buffer and glucose curves demonstrates excellent catalytic performance and strong interaction between the enzyme and the conductive matrix. Overall, all four composites exhibit a detectable electrochemical response to glucose. However, the magnitude of this response varies depending on the nanomaterial used, with PANI@CD and PANI@MWCNT-ZnO showing the highest current at 800 mV, followed by PANI@BGr, while PANI@MWCNT-COOH demonstrates relatively weaker activity.

### Analytical response of the electrochemical biosensor

#### Calibration in buffer for different allotropes of carbon-based composites

**Fig. 6:**
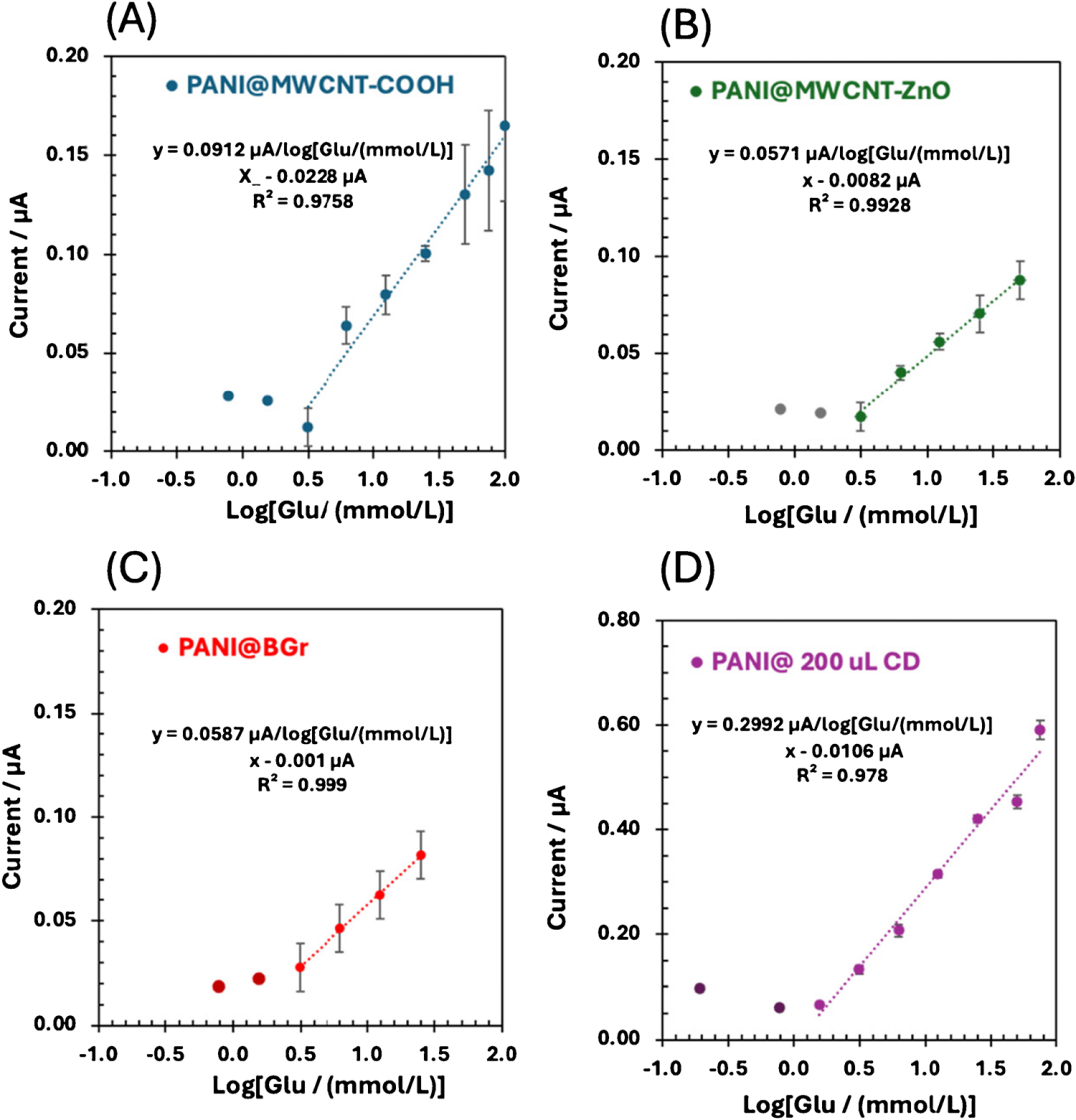
Calibration curve of glucose concentrations for different composites: **a** PANI@MWCNT-COOH; **b** PANI@MWCNT-ZnO; **c** PANI@Bgr; and **d** PANI@CD. The calibration curves represent the current response against the logarithmic glucose concentration for the time of 180 s

Calibration curves were generated in PBS buffer under physiological conditions to determine the relationship between the measured current and the logarithm of the glucose concentration for biosensors produced with different composites: (i) PANI@MWCNT-COOH; (ii) PANI@MWCNT-ZnO; (iii) PANI@CD; and (iv) PANI@BGr. Current values were recorded after 180 s using the CA technique. The glucose concentrations used for calibration were 0.19 to 100 mmol/L for PANI@CD and 0.78 to 100 mmol/L for PANI@BGr, PANI@MWCNT-COOH, and PANI@MWCNT-ZnO (Fig. 6).

The analytical performance of each sensor was compared based on the slope of the calibration curve, the coefficient of correlation (*R*^2^), the lower limit of the linear range (LLLR), the upper limit of the linear range (ULLR), and the LOD. The PANI sensor showed a slope of 0.081 μA/log[glu/(mmol/L)] and an *R*^2^ of 0.982, indicating good linearity. It had an LLLR of 1.56 mmol/L, a ULLR of 50.0 mmol/L, and an LOD of 1.65 mmol/L, reflecting good sensitivity and a satisfactory working range (Figure S1).

Modification with carbon nanotubes (PANI@MWCNT-COOH) increased the slope to 0.091 μA/log[glu/(mmol/L)], suggesting slightly improved sensitivity. However, the LLLR increased to 3.13 mmol/L and the LOD increased to 2.04 mmol/L, indicating a reduced ability to detect very low glucose concentrations. In contrast, the ULLR extended to 100 mmol/L, the widest range among the sensors (Fig. 6a).

The PANI@MWCNT-ZnO sensor exhibited a lower slope of 0.057 μA/log[glu/(mmol/L)], but the highest *R*^2^ value (0.993), confirming excellent linearity. The LLLR was 3.13 mmol/L, LOD 2.67 mmol/L while the ULLR reached 50.0 mmol/L (Fig. 6b). Incorporation of BGr (PANI@BGr) further reduced the slope to 0.051 μA/log[glu/(mmol/L)], with an *R*^2^ of 0.999. Despite the lower sensitivity, this sensor maintained an LLLR of 3.13 mmol/L, LOD of 3.04 mmol/L, and an ULLR of 25.0 mmol/L, remaining effective for glucose detection at low concentrations (Fig. 6c). Finally, the PANI@CD sensor exhibited the highest slope among all, 0.299 μA/log[glu/(mmol/L)], indicating superior sensitivity. The *R*^2^ value of 0.978 confirmed good linearity, while the LOD was 1.08 mmol/L, with an LLLR of 1.56 mmol/L and a ULLR of 75.0 mmol/L (Fig. 6d). These results make it one of the most balanced sensors in terms of both sensitivity and working range. Overall, the choice of nanostructured modifiers significantly affects the analytical response of the sensors. The PANI@CD composite proved to be the most promising due to its notably higher sensitivity, while the PANI@MWCNT-COOH sensor offered the broadest linearity range.

Overall, in the PANI@CD composite, CDs outperform other allotropes due to their unique interfacial and electron transfer, surface area, morphology, and material origin. The CDs synthesized from fructose have abundant oxygen-containing functional groups (–OH, –COOH), which facilitate strong enzyme immobilization and optimal orientation for electron transfer [8]. This was confirmed by FTIR analysis (Figure S2). The FTIR spectrum of the CDs shows multiple oxygen-containing surface functional groups, confirming their hydrophilic property. A broad absorption band centered at approximately 3338 cm⁻^1^ is assigned to O–H stretching vibrations, originating from surface hydroxyl groups and/or physisorbed water molecules on the CD surface. The extent of this band also indicates extensive hydrogen-bonding interactions. The band at 2884 cm⁻^1^ corresponds to C–H stretching vibrations associated with aliphatic carbon frameworks. In the fingerprint region, the absorption peak at around 1251 cm⁻^1^ is attributed to C–O or C–O–C stretching vibrations, indicating alcohol or epoxy functionalities. The prominent band near 1129 cm⁻^1^ further supports the presence of C–O stretching modes from oxygenated surface groups. Overall, the high density of oxygen-containing functionalities evidenced by the FTIR analysis suggests that the CDs have enhanced surface reactivity and good dispersibility in aqueous environments (Figure S2).

Second, their nanoscale, quasi-spherical morphology ensures a high density of electroactive sites and a large effective surface area, minimizing aggregation compared to MWCNTs or BGr sheets (see Fig. 2). Third, the combination with PANI creates a network in which CDs act as electron bridges, reducing charge-transfer resistance and promoting faster glucose oxidation at the electrode interface.

#### Effect of the amount of CD in the analytical performance

**Fig. 7:**
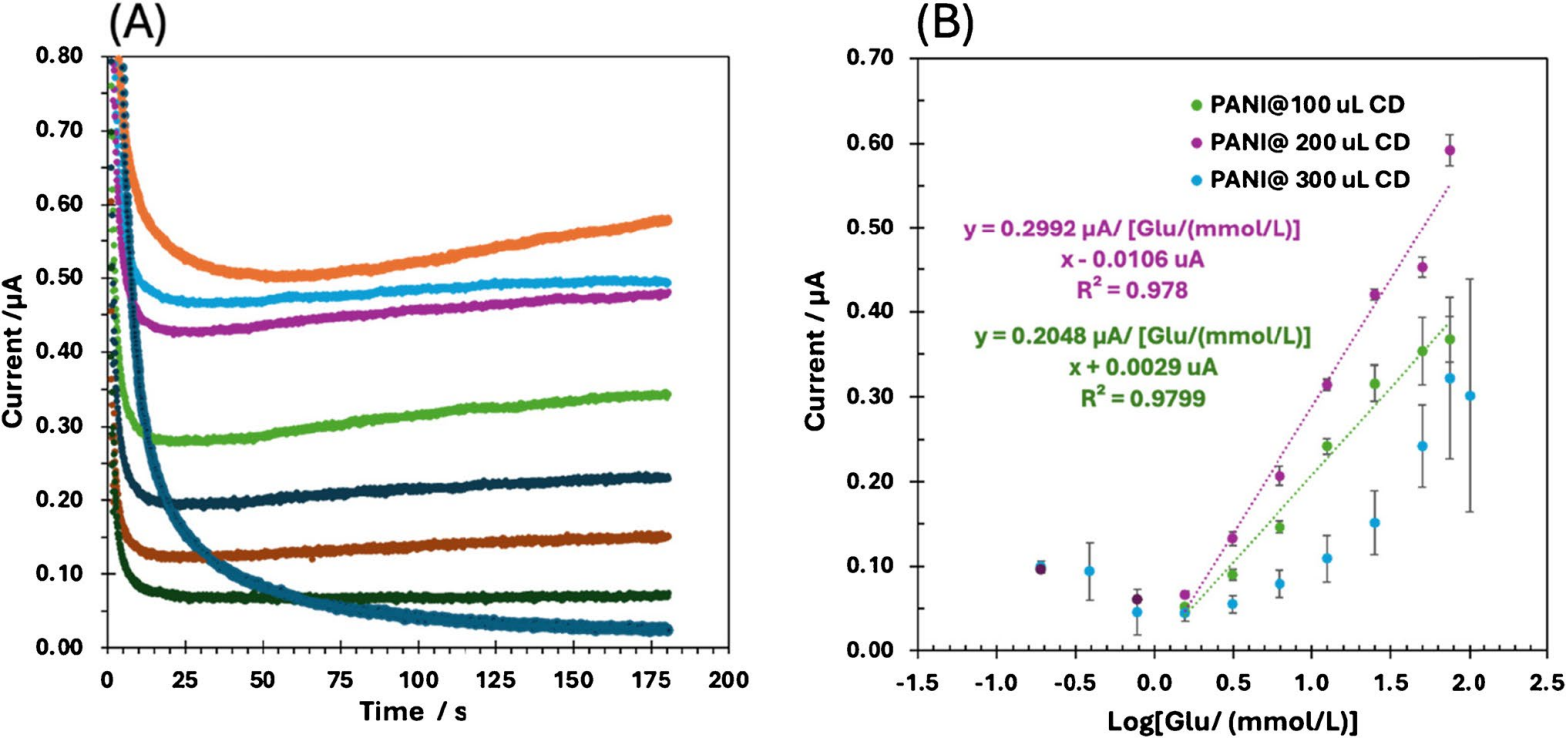
CA measurements (**A**) and the respective calibration curves (**B**) obtained for synthesis of the PANI@CD with different concentrations of CD prepared in PBS buffer pH 7.2

Given the excellent operating characteristics of the PANI@CD composite, it demonstrated superior analytical performance, particularly regarding slope. Figure 7a presents the CA spectra for calibration using a 20-fold CD dilution, while Fig. 7b shows the calibration curves for the various CD dilutions tested.

To optimize sensor performance, various CD concentrations were evaluated, including 5-fold, 10-fold, and 20-fold dilutions. The 10-fold dilution (200 µL) produced the highest slope, 0.2992 μA/log[glu/(mmol/L)], surpassing all other dilutions. This result demonstrates significantly improved sensitivity. The corresponding *R*^2^ value of 0.978 indicates excellent linearity, which is essential for reliable analytical measurements. Additionally, this dilution showed an LLLR of 1.56 mmol/L and an LOD of 1.08 mmol/L, enabling detection of low concentrations, as well as a ULLR of 75.0 mmol/L, indicating a wide dynamic range. These results highlight the critical importance of the 10-fold dilution for optimal sensor performance.

#### Calibration in serum

**Fig. 8:**
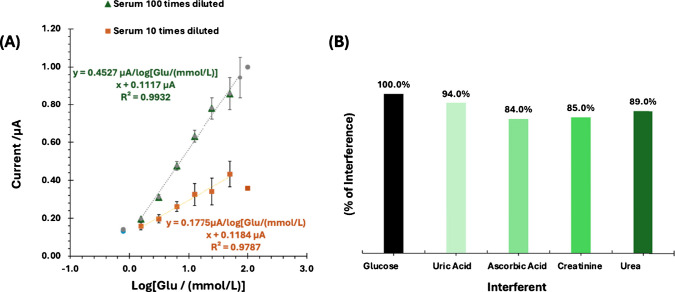
**A** Calibration curve of glucose concentrations (0.78 to 100 mmol/L) in spiked serum samples with different dilutions of serum 10-fold and 100-fold, respectively. **B** Relative current intensity data extracted from the CA plots for glucose (5.00 mmol/L) and glucose (5.0 mmol/L) mixed with uric acid (0.416 mmol/L); ascorbic acid (0.085 mmol/L); creatinine (0.115 mmol/L); and urea (6.662 mmol/L)

Calibration curves were generated in serum diluted 10-fold and 100-fold in PBS buffer under physiological conditions to determine the relationship between the measured current and the logarithm of the glucose concentration for the sensor with PANI and CD (200 µL). Current values were recorded after 180 s using the CA technique. Glucose concentrations for calibration ranged from 1.56 to 100.0 mmol/L (Fig. 8A). A higher slope of 0.4623 μA/log[glu/(mmol/L)] was observed for the 100-fold diluted serum, while the ten-fold diluted serum showed a slope of 0.1775 μA/log[glu/(mmol/L)]. Both dilutions had a LLLR starting at 1.56 mmol/L and a LOD of 1.08 mmol/L. These results indicate that the sensor has higher sensitivity in serum at higher dilutions, likely due to interactions between the sensor and the complex biomolecules present in the serum.

When comparing these results with the calibration performed in buffer, a significant increase in the slope was observed to be approximately twice as high at the 100-fold dilution of serum. This significant improvement in sensitivity can be attributed to the more complex and dynamic composition of serum compared to the simple PBS buffer. Serum contains a variety of proteins, ions, and other biological molecules that interact with the sensor surface and enhance its electrochemical response. These interactions improve not only the current response but also the overall performance of the sensor, as indicated by the higher slope values observed in serum. Additionally, the greater slope in serum calibration highlights the importance of performing biosensor calibrations under conditions that closely mimic real biological environments. Calibration in buffer solutions cannot fully replicate the challenges or advantages a biosensor encounters in a biological system, making analysis in serum essential for accurate, real-world applications. Serum calibration results ensure that the biosensor’s performance is validated under conditions similar to those in clinical or diagnostic scenarios, leading to more reliable and meaningful measurements.

Figure 8b shows the results of a selectivity study on a glucose sensor, evaluating its response to glucose in the presence of potential interfering substances. The mixed solutions method was used to determine the percentage of interference, with the current measured for glucose at 5.0 mmol/L serving as the reference signal, set at 100%. The study assessed the effects of uric acid (0.416 mmol/L), ascorbic acid (0.085 mmol/L), creatinine (0.115 mmol/L), and urea (6.662 mmol/L) on glucose detection. In the absence of interferents, the sensor’s response to glucose was defined as 100%. When glucose was mixed with uric acid, the sensor signal decreased by 6%, resulting in 94% relative to the glucose-only signal. Ascorbic acid, creatinine, and urea caused signal reductions of 16%, 15%, and 11%, respectively, relative to the glucose baseline. These results show that all tested interferents slightly affect the sensor’s glucose detection, with the most pronounced interference observed for ascorbic acid (16%), creatinine (15%), and uric acid (6%). Overall, the sensor demonstrated good selectivity, maintaining a high response even in the presence of uric acid and urea, with interference values of 6% and 11%, respectively.

Table S1 provides an overview of several commercially available biosensing devices for glucose and cholesterol monitoring, including their analytical ranges, sample volume requirements, and target analytes. Most commercial glucose biosensors operate over concentration ranges of approximately 0.6–33.3 mmol/L and require minimal blood volumes, typically below 1 µL, which highlights their suitability for rapid point-of-care testing. In contrast, cholesterol monitoring systems generally require larger sample volumes, typically 4–40 µL of blood, reflecting the greater complexity of lipid analysis. Compared to these commercial platforms, the biosensor developed in this study offers an expanded glucose detection range of 1.56–75.0 mmol/L, demonstrating its ability to quantify both normoglycemic and markedly hyperglycemic concentrations. Although the proposed platform requires a higher sample volume of 30 µL serum, its extended analytical range underscores its potential for clinical monitoring and biosensing applications that require broader detection capabilities.

## Conclusions

This study demonstrates the development of a versatile, high-performance electrochemical glucose biosensor platform by integrating PANI with various carbon allotropes, such as BGr, MWCNT-COOH, MWCNT-ZnO, and CDs. Each composite exhibited unique structural and electrochemical properties that directly influenced the biosensor’s performance. Among all tested materials, the PANI@CD-based biosensor showed the most promising analytical behavior, presenting the highest sensitivity with a slope of 0.2999 μA/log[glu/(mmol/L)] in buffer and up to 0.4623 μA/log[glu/(mmol/L)] in serum, as well as the lowest limit of the linear range at 1.56 mmol/L. The analytical performance of the biosensor is comparable to that of commercially available biosensing devices (Table S2).

A particularly remarkable result of this work is the use of CD synthesized from biologically derived compounds, specifically fructose and manganese sulfate, through a simple, low-cost microwave-assisted method. The biogenic origin of the CDs provides enhanced surface functionality rich in oxygen-containing groups, as confirmed by XPS analysis. These functional groups improve the electrochemical activity of the sensor interface, facilitate stronger enzyme immobilization, and enable efficient electron transfer during glucose oxidation.

In addition to their structural and functional advantages, the CDs provide inherent biocompatibility, environmentally friendly synthesis, and scalability, which are important properties for developing sustainable and clinically useful sensor platforms. Their integration into the biosensor matrix represents a strategic improvement that makes green nanotechnology suitable for biomedical applications. The significant enhancement in sensor performance achieved with PANI@CD composites highlights the crucial role of bio-derived carbon nanomaterials in advancing next-generation enzymatic biosensors. Overall, this work not only identifies carbon dots as the optimal modifier among the investigated carbon allotropes but also underscores the broader potential of bio-derived nanomaterials for fabricating sensitive, selective, and sustainable biosensors. These results open new opportunities for developing high-performance electrochemical platforms suitable for real-time glucose monitoring in clinical diagnostics and personalized medicine.

## Supplementary Information

Below is the link to the electronic supplementary material.

Supplementary file1 (DOCX 210 KB)

## Data Availability

All data generated or analyzed during this study are included in this article.
