# Supplementary material for: Effect of different carbon allotropes for electrochemical glucose detection
Source: Anal Bioanal Chem. 2026 Jun 6;418(15):4915–28. doi: 10.1007/s00216-026-06595-y (PMC13388455; doi:10.1007/s00216-026-06595-y)
Supplement: Supplementary file 1 — Supplementary file1 (DOCX 210 KB) [file 216_2026_6595_MOESM1_ESM.docx]

Supplementary information

Effect of Different Carbon Allotropes for electrochemical glucose detection

Rui Couto^1^, Joaquim Alves^1^, Abel Duarte^1^, Stefano Chiussi^2^, Cristina Sousa^1^ and Felismina T.C. Moreira

***Figure S1.*** *Calibration curve of glucose concentrations for PANI based sensor*

Figure S2 - FTIR spectra of carbon dots.

**Table S1 -** Commercially available biosensing devices.

| **Device / Sensor** | **Concentration Range (mmol/L)** | **Sample Requirement** | **Analyte** | **References** |
| --- | --- | --- | --- | --- |
| Accu-Chek Guide | ~0.6–30.0 | ~0.6 µL blood | Glucose | (1) |
| OneTouch Ultra 2 | ~0.6–33.3 | Small blood sample | Glucose | (2) |
| FreeStyle Lite | ~0.6–33.3 | Fingerstick blood | Glucose | (3) |
| Contour Next ONE | ~0.6–33.3 | Small sample | Glucose | (4) |
| GlucoRx Nexus | ~1.1–30.0 | ~0.5 µL blood | Glucose | (5) |
| TRUE METRIX PRO | ~1.1–33.3 | Small blood sample | Glucose | (6) |
| Contour Next EZ | ~0.6–33.3 | Small sample | Glucose | (7) |
| Accutrend Plus | Cholesterol: ~3.88–7.76 | ~15–40 µL capillary blood | Cholesterol, triglycerides, lactate, glucose | (8) |
| CardioChek PA | Cholesterol: ~2.59–10.35 | ~15–40 µL fingerstick blood | Lipid panel (TC, HDL, TG), glucose | (9) |
| Mission Cholesterol | Cholesterol: ~2.59–10.35 | ~4 µL blood | Total cholesterol | (10) |
| BeneCheck Plus | Cholesterol: ~2.59–10.35 | ~10 µL blood | Glucose, cholesterol, uric acid | (11) |
| This research | 1.56 to 75.0 | 30 µL serum | glucose |  |

1. Roche Diabetes Care. *Accu-Chek Guide blood g*lucose monitoring system user manual. Mannheim: Roche Diabetes Care GmbH 2020 <https://www.accu-chek.com/meters/accu-chek-guide>.

2. LifeScan Inc. *OneTouch Ultra 2 blood gluco*se monitoring system owner’s booklet. Malvern, PA: LifeScan 2020 <https://www.onetouch.com/products/onetouch-ultra2>.

3. Abbott Diabetes Care. *FreeStyle Lite blood glu*cose monitoring system user’s manual. Alameda, CA: Abbott Diabetes Care Inc 2020<https://www.freestyle.abbott/us-en/home.html>.

4. Ascensia Diabetes Care. CONTOUR NEXT ONE user guide. Parsippany, NJ: Ascensia Diabetes Care; 2020 <https://www.contournextone.com/>.

5. GlucoRx Ltd. *GlucoRx Nexus blood g*lucose monitoring system user manual. Surrey: GlucoRx Ltd. 2019 <https://www.glucorx.co.uk/products/nexus-meter>.

6. Trividia Health Inc. *TRUE METRIX PRO professional* monitoring system instructions for use. Fort Lauderdale, FL: Trividia Health Inc.; 2018 <https://www.trividiahealth.com/true-metrix-pro>.

7. Ascensia Diabetes Care. CONTOUR NEXT EZ user guide. Parsippany, NJ: Ascensia Diabetes Care; 2019 [Available from: <https://www.ascensiadiabetes.com/>.

8. Roche Accutrend® Plus system 2019 <https://diagnostics.roche.com/global/en/about/innovation.html>.

9. CardioChek PA Analyzer 2019 [Available from: <https://www.ptsdiagnostics.com/cardiochek-pa-analyzer/>.

10. Mission® Cholesterol – 2019 [Available from: <https://www.aconlabs.com/brands/mission/mission-cholesterol/>.

11. BeneCheck PLUS Multi‑Monitoring System 2019 [Available from: <https://www.manualslib.com/manual/1027140/Benecheck-Plus-Series.html>.
